# Honest, Open, Proud to support disclosure decisions and to decrease stigma’s impact among people with mental illness: conceptual review and meta-analysis of program efficacy

**DOI:** 10.1007/s00127-021-02076-y

**Published:** 2021-04-24

**Authors:** Nicolas Rüsch, Markus Kösters

**Affiliations:** Department of Psychiatry II, Ulm University and BKH Günzburg, Ulm/Günzburg, Germany

**Keywords:** Stigma, Self-stigma, Stigma stress, Disclosure, Honest, Open, Proud, Coming Out Proud, Peer intervention, Meta-analysis

## Abstract

**Purpose:**

Honest, Open, Proud (HOP; formerly “Coming Out Proud”/COP) is a peer-led group program to support people with mental illness in their disclosure decisions and in their coping with stigma. The aims of this study were to provide (i) a conceptual review of HOP, including versions for different target groups and issues related to outcome measurement and implementation; and (ii) a meta-analysis of program efficacy.

**Methods:**

Conceptual and empirical literature on disclosure and the HOP program was reviewed. Controlled trials of HOP/COP were searched in literature databases. A meta-analysis of HOP efficacy in terms of key outcomes was conducted.

**Results:**

HOP program adaptations for different target groups (e.g. parents of children with mental illness; veterans or active soldiers with mental illness) exist and await evaluation. Recruitment for trials and program implementation may be challenging. A meta-analysis of five HOP RCTs for adults or adolescents with mental illness or adult survivors of suicide attempts found significant positive effects on stigma stress (smd = − 0.50) as well as smaller, statistically non-significant effects on self-stigma (smd = − 0.17) and depression (smd = − 0.11) at the end of the HOP program. At 3- to 4-week follow-up, there was a modest, not statistically significant effect on stigma stress (smd = − 0.40, 95%-CI -0.83 to 0.04), while effects for self-stigma were small and significant (smd = − 0.24). Long-term effects of the HOP program are unknown.

**Conclusion:**

There is initial evidence that HOP effectively supports people with mental illness in their disclosure decisions and in their coping with stigma. Implementation issues, future developments and public health implications are discussed.

## Introduction

People with mental illness face the symptoms of their disorder as well as stigma and discrimination. The consequences for labeled individuals are significant and include status loss [1], disadvantages with respect to housing [2], work [3] or education, social isolation, poor health care and suicidality [4, 5]. Many people with mental illness are not only aware of public prejudices, but turn them against themselves which is referred to as self-stigma (“I have a mental illness, therefore I must be lazy” [6]). Self-stigma is associated with a range of negative outcomes [7] and leads to impaired self-esteem and a sense of demoralization, the so-called why try effect (“Why should I even try to achieve my goals? I am not worthy or not able” [8]). In longitudinal studies, self-stigma was a predictor of increased symptoms and of suicidality [7]. Stigma can also be a stressor if people with mental illness perceive stigma-related harm to be higher than their coping resources; this so-called stigma stress predicts self-stigma [5], poor quality of life, higher symptom levels and worse clinical outcomes [9, 10].

In this paper, which builds on previous work of Pat Corrigan and of our group [11, 12], we will first outline existing approaches to reduce self-stigma, discuss the identity of being “mentally ill” and the role of disclosure decisions. We then present approach and content of the peer-led group program “Honest, Open, Proud” (HOP) for people with mental illness that focuses on disclosure decisions and offers a novel way to reduce self-stigma and stigma’s impact, followed by an overview of current issues related to program delivery and a meta-analysis of HOP efficacy.

### Approaches to reduce self-stigma

There are a range of programs to reduce self-stigma [13, 14]. In psychoeducational interventions, such as Ending Self-Stigma developed by Patricia Lucksted and Amy Drapalski, participants learn facts that contradict their self-stigmatizing beliefs. However, there is limited evidence for the efficacy of this approach [15, 16]. Cognitive therapies aim to correct self-stigma as a distorted self-concept, but their benefit is unclear [17]. Acceptance-based approaches use principles of acceptance and commitment therapy and mindfulness to reduce self-stigma and improve self-esteem [18]. Narrative programs help participants to develop more balanced stories of their lives; Narrative Enhancement and Cognitive Therapy, developed by Paul Lysaker, David Roe and Phil Yanos, is an example of a comprehensive narrative-cognitive strategy that reduces self-stigma [19]. Another approach to reduce self-stigma is to support people with mental illness in their disclosure decisions—this is the focus of the HOP program which will be discussed in more detail below. Mental illness being a discreditable stigma, as Erving Goffman put it [20], many face the decision whether and how to disclose their condition to others, and (non-) disclosure is a key reaction to stigma.

A recent meta-analysis examined the efficacy of interventions to reduce self-stigma across 14 controlled trials [13]; the majority of included studies investigated psychoeducational (five) or narrative (three) programs. On average, results were positive for psychoeducational and narrative interventions despite the small number of studies that were in part of poor quality. Findings were inconclusive for cognitive interventions. The conclusions of this meta-analysis were limited by the fact that it did not include all RCTs of interventions to reduce self-stigma published at that time [21].

Independent of the intervention strategy, self-stigma is nobody’s fault nor is it a clinical problem, although clinicians should be aware of it as it can be a major barrier to service use, shared decision making and recovery [22–24]. Self-stigma is a consequence of public stigma and interventions should support people with mental illness to cope with self-stigma as long as public stigma exists.

### Identity of being “mentally ill”

People with mental illness can react very differently to a psychiatric diagnosis. They may perceive it as a helpful concept or term that facilitates access to care and communication with clinicians. But diagnoses can also be seen as a stigmatizing label that attributes deficits to the person and reduces individuals to an illness without conveying a sense of hope [25]. There is the apparent paradox that insight, i.e. a greater awareness of having a mental illness, can be associated with better and with worse clinical or social outcomes. Paul Lysaker and colleagues found that if people with schizophrenia reject stigma as unfair, insight does not affect them negatively; however, if they suffer from self-stigma and agree with negative stereotypes about their group, greater insight leads to hopelessness [26]. Thus for an individual, to have an identity or self-concept as a person with mental illness is not bad or good per se—it depends on what the person associates with this identity.

### Disclosure

People with mental illness often face the difficult choice whether to disclose their condition. Similar to the identity of having a mental illness, disclosure or non-disclosure is not good or bad per se—their consequences depend on the person and the environment [27]. In stigmatizing environments, the risks of disclosure often outweigh the benefits. A longitudinal study among long-term unemployed individuals that adjusted for clinical, work-related and socio-demographic variables provides an example: Those who were more willing to disclose their mental illness during their job search were *less* likely to be re-employed six months later [3]. However, greater comfort with disclosing to family and friends at baseline led to *better* quality of life over time [28]. All in all, the evidence suggests that disclosing one’s mental illness improves well-being and social support [27]. However, disclosure decisions remain personal and complex because contexts and personal vulnerabilities differ: For some, not all, individuals secrecy leads to ruminations [27] and they may feel proud communicating their lived experience of mental illness and experiencing the authenticity that comes with disclosure [12]. Therefore, disclosure decisions are a key reaction to public and self-stigma and can affect self-stigma, stigma stress and well-being.

### Approach and aim of Honest, Open, Proud

Considering the above-mentioned complexities of identity and disclosure and following previous work [29, 30], Corrigan and his colleagues in Chicago developed HOP as a peer-led group program to help participants decide whether and how to disclose their mental illness. HOP’s focus on disclosure decisions is based on models that highlight the role of (non-)disclosure for coping with the stigma associated with a concealable stigmatized identity [31, 32] and on findings that concealment versus disclosure is a dilemma for many people with mental illness [33]. These models highlight that disclosure decisions are a key component of successful coping with stigma and thus a mechanism to reduce stigma stress, self-stigma and their consequences, such as depression and reduced well-being or quality of life [34].

It is not HOP’s goal to push people towards disclosure (although the program’s English name has been misunderstood to suggest this). Non-disclosure in a stigmatizing environment can be a perfectly reasonable choice. HOP can lead to careful disclosure as well as to non-disclosure that is not driven by self-stigma or shame. HOP facilitator Chris White, Glasgow, called the latter outcome “empowered non-disclosure” (personal communication).

### HOP content

HOP is a manualized program and consists of three lessons. Often HOP is therefore delivered in three two-hour sessions, with one session per week. Current HOP versions include a fourth booster session about a month after the third lesson.

In Lesson 1, participants first discuss how central mental illness is to their identity as well as possible reasons for disclosure (e.g. to find social support, to increase one’s authenticity). The main part of Lesson 1 deals with benefits and risks of (non-)disclosure. Participants define their goals and what they expect after (non-)disclosure. At the end of the lesson, participants should be able to make a preliminary decision for or against disclosure in a certain setting.

Lesson 2 covers five levels of disclosure. Secrecy and disclosure are not all-or-nothing choices, but lie on a continuum between the extremes of social withdrawal (to avoid that others find out about one’s mental illness) and active broadcasting of one’s lived experience. In between are secrecy or non-disclosure (without social withdrawal), selective disclosure (of certain information to selected individuals), and indiscriminate disclosure (without active broadcasting). Lesson 2 also helps to prepare disclosure decisions. Participants learn how to “test the waters” with potential addressees of their disclosure. A participant could, outside the HOP group session, talk to a colleague about a TV series she saw last night that portrayed a person with mental illness. If the colleague responds with stigmatizing comments, she will be warned to be careful with disclosing to this colleague.

Lesson 3 develops ways to tell one’s story. The workbook offers an outline to develop one’s story that includes experiences with the (beginning) mental illness, dark days, recovery and experiences how to overcome stigma and discrimination. Lesson 3 ends by reflecting on what participants have learnt in the program and how peer support can help them to move on. In Lesson 4, as a booster session, participants recall their attitudes towards disclosure at the end of Lesson 3 and discuss their experiences with (non-)disclosure in the meantime, incl. whether that has changed their choices regarding disclosure or the stories they would like to tell.

### Aims of this study

There are a number of recent systematic reviews on interventions to reduce self-stigma among people with mental illness [13, 14, 35–37]. Support with disclosure decisions is a novel strategy to reduce self-stigma. The approach has been outlined in conceptual work on HOP [12], and shorter pieces have commented on different aspects of HOP [38, 39]. However, we are not aware of a review or meta-analysis that specifically deals with HOP’s efficacy, and the most recent review of programs to reduce self-stigma [13] was incomplete with regard to published HOP trials [21]. A synthesis of available evidence is therefore needed to guide future work on HOP’s evaluation and implementation.

This paper had two aims, (i) to provide a conceptual narrative review on the HOP program incl. issues related to different versions and program implementation; and (ii) to provide a meta-analysis on the efficacy of the HOP program based on controlled trials. Both aims are interconnected: In case the meta-analysis provides evidence that HOP is beneficial, issues of program delivery in different settings become more important (Table 1).

**Table 1:** Versions of the HOP program for specific target groups; versions with different names than HOP add “…—a HOP program” to their name (e.g. “2Share—a HOP program” to clarify that 2Share is a HOP version)

Target group	Program name	Lead program developers	Evaluations
Adults with mental illness	*HOP* (the original standard version from which other versions are derived)	Patrick W. Corrigan, Chicago, US	2 RCTs [47, 48], 1 feasibility study [51]
Adolescents with mental illness	*HOP* or *Up to Me*	Sarah Reed, Sue McKenzie & Suzette Urbashich, Wisconsin, and Pat Corrigan, Chicago, US	1 RCT [21]
College students with mental illness	*HOP-College*	Maya Al-khouja and P.W. Corrigan, Chicago, US	1 RCT [49]
Parents of children with mental health challenges	*Starting the Conversation*	Jeneva Ohan, Perth, Australia	–
Suicide attempt survivors	*2Share*	Lindsay Sheehan, Chicago, US	1 RCT [46]
Soldiers with mental illness	*HOP*	Nicolas Rüsch, Ulm, Germany	Pilot RCT ongoing^a^
Veterans with mental illness	*HOP*	Michelle Andra, Veterans’ Health Administration, and Jon Larson, Chicago, US	–
Mental health professionals with mental illness	*HOP-MHP* (delivered as anonymous online self-help guide)	Katrina Scior, London, UK	Pilot RCT completed^b^
People with Tourette Syndrome	*HOP: To Eliminate the Stigma of Tourette*	Sarah Reed, Sue McKenzie & Ellie Jarvie, Wisconsin, US	–
People with substance use disorders	*HOP-SUD*	Patrick W. Corrigan, Chicago, US	–
People with urinary incontinence	*To Tell or Not to Tell*	Lindsay Sheehan, Chicago, US	–
People with dementia and their significant others	*Who to Tell, How and When?*	Jemini Bhatt, London, UK	1 feasibility study [50]
People with psychosis	*Let’s talk* (delivered as 1-to-1 peer support)	Melissa Pyle, Manchester, UK	Pilot RCT ongoing^c^

^a^https://clinicaltrials.gov/ct2/show/NCT03218748

^b^http://www.isrctn.com/ISRCTN18418155

^c^https://www.fundingawards.nihr.ac.uk/award/NIHR200460

## Methods

First, we non-systematically surveyed conceptual and empirical literature to inform our narrative review on disclosure, mental illness and the HOP intervention, incl. HOP training materials and trial registries for ongoing HOP trials. Second, we searched for controlled trials of the HOP program (previously known as COP/Coming Out Proud) program, randomized or not randomized, in Pubmed, Medline and PsycInfo on November 15, 2020, and again on February 9, 2021, for our meta-analysis. “Honest, Open, Proud”, “Coming Out Proud”, and “In Würde zu sich stehen” (IWS, the program’s German name) were used as search terms. English and German language articles were searched without time limit. Reference lists of trial publications were checked, and colleagues active in HOP research were contacted. All controlled trials of COP or HOP were included in the meta-analysis while uncontrolled pre-post evaluations were not. The search was run by NR and checked by MK; data were extracted from RCT datasets by NR and discussed with MK. We extracted data on the above-mentioned outcomes (Table 2) as well as on study and sample characteristics (Table 3) and, if available, on feasibility and implementation.

**Table 2:** Selected key outcome measures in published HOP RCTs

Measure	Domain	Number of items	Range of scale scores (mean or sum)	HOP RCTs that reported this outcome	Original publication of the scale
Stigma Stress Scale (SSS)	Stigma stress (i.e., cognitive appraisal of stigma as a stressor)	8	− 6 to + 6 (difference score, harm/subscale 1 minus coping resources/subscale 2), higher score = more stigma stress	[21, 46, 47]	[57]
SSS subscale 1: perceived harm	Appraisal of stigma as harmful	4	1–7 (mean), higher score = more stigma-related harm	[46, 48, 49]	(As above)
SSS subscale 2: perceived coping resources	Appraisal of resources to cope with stigma	4	1–7 (mean), higher score = more coping resources	[46, 48, 49]	(As above)
Self-Stigma of Mental Illness Scale-Short Form (SSMIS-SF)	Stepwise process of self-stigma (aware, agree, apply, harm)	20 (4 subscales with 5 items each)	1–9 mean score for each subscale (no total score across subscales), higher score = more awareness, agreement etc.	All subscales [48, 49]; Apply subscale [21]; Harm subscale [46]	[58]
Internalized Stigma of Mental Illness Inventory (ISMI)	Broad self-stigma measure, incl. experienced discrimination and social withdrawal	29 (24, if without the 5 stigma resistance items)	1–4 (mean), higher score = more self-stigma	[47]; version for suicide self-stigma [46]	[59]
Internalized Stigma of Mental Illness Inventory-Short Form (ISMI-SF)	(same as long version)	10	1–4 (mean), higher score = more self-stigma	[21]	[60]
Center for Epidemiological Studies-Depression Scale (CES-D)	Depressive symptoms	10 (English short version); 15 (German short version)	0–3 (mean), higher score = more depressive symptoms	[21, 48, 49]	English [61]; German [62]
KIDSCREEN-10 index	Health-related quality of life	10	10–50 (sum), higher score = better quality of life	[21]	[63]
General Help-Seeking Questionnaire (GHSQ)	Intentions to seek help from professionals (subscale 1) or family/friends (subscale 2)	4 items in each subscale	1–7 (mean) for each subscale, higher score = stronger intention to seek help	[21]	[64]
Self-Identified Stage of Recovery (SISR)	Stage of recovery (part A); recovery processes (part B)	Part A: 1 stage out of 5; Part B: 4 items	Stage 1–5 (part A); 1–6 (mean, part B). Higher scores = more recovery	Part B [21]	[65]

### Meta-analysis

We selected three outcome domains for the meta-analysis: stigma stress, self-stigma, and depressive symptoms. For each outcome domain, we planned to choose one measure, if possible, that was used in all trials. Effect sizes were calculated as standardized mean differences (Hedges’ g), comparing the means of the HOP intervention group to the treatment as usual group at post intervention and follow-up assessments. For the purpose of this meta-analysis, principal investigators of HOP trials gave us access to the individual participant data of all included trials. For data that had not been published or for outcomes that had been reported with different scorings (e.g. sum scores instead of mean scores), we computed the mean scores for stigma stress, depression and self-stigma from the RCT data sets such that each outcome was on the same scale and could be compared across trials.

Sometimes change scores from baseline rather than post-baseline scores are used to account for baseline differences between intervention and control groups or for problems with randomization. However, this approach is not recommended [40] and less conservative [41], therefore our meta-analysis is based on post-baseline scores. Effect sizes are considered small in the range of 0.2, medium at about 0.5 and large at values of 0.8. Due to differences between samples or study designs, statistical heterogeneity of effect sizes had to be expected. Therefore, effect sizes were combined within a random effects model. Heterogeneity of effect sizes was assessed with the *I*^2^ parameter. *I*^2^ is a descriptive statistic of the percentage of the variability in effect estimates that is due to heterogeneity rather than chance; the higher the *I*^2^ values from 0 to 100%, the higher the heterogeneity. All analyses were conducted with RevMan 5 [42].

## Results

### HOP versions for different target groups

The original HOP version for adults with mental illness has been adapted for different cultural contexts and target groups (Table 1). In the spirit of participatory research, HOP was adapted in cooperation between the target group and other relevant stakeholders because stigma varies between cultures and different conditions [39]. For example, to create a German HOP version for adolescents with mental illness, the adolescent version developed in the US was first translated. Based on focus groups and discussions with adolescents with and without mental illness, teachers, parents and mental healthcare providers, the content was then culturally adapted [21, 43, 44]. Information about HOP versions is available in English (hopprogram.org) and German (uni-ulm.de/med/iws/) and from lead developers listed in Table 1.

### Group facilitators, participants, program fidelity

#### HOP group facilitators

HOP groups are facilitated by peers that is by people with lived experience of mental illness. This lived experience makes group facilitators credible for participants. It is also helpful for group facilitators to share a specific background that affects disclosure decisions. For example, in a current trial of HOP for active soldiers in Germany, groups are facilitated by soldiers with mental illness who understand the specific pros and cons of disclosing in the military. Regarding the choice of group facilitators, there is a degree of flexibility. In our trial of HOP for adolescents in inpatient settings, groups were run by a young adult peer and a young mental health professional [21]. Regarding the number of group facilitators, two peers often run a group together. Group facilitators are usually trained in a two-day workshop, followed by supervision as needed.

#### HOP participants

It is not necessary that participants have received a psychiatric diagnosis or treatment although this is usually the case. They should have personal experience with mental health problems and have dealt with disclosure decisions. Groups ideally consist of four to eight participants. If the number is higher or smaller, a lively exchange between group members can be difficult. Finally, participants should not join the group after the first session.

#### Fidelity

In the RCTs, program fidelity was assessed by a checklist. Research assistants sat in the background of each session and noted whether key content, e.g. a worksheet with group discussion, had been covered in each lesson. Fidelity was high in the range of 80–90%. Outside the research context, it is a challenge to make the program available and boost its implementation while maintaining satisfactory quality of program delivery. A current Australian study addresses this question with an implementation science framework and collects qualitative and quantitative data to assess the feasibility of implementing HOP [45]. With respect to preparing peers for running groups, HOP follows a train-the-trainer approach: Interested individuals are trained in centers with HOP expertise. Trained group facilitators can then run HOP groups and learn from the exchange with other group facilitators as well as from HOP master trainers as supervisors.

### HOP trials

Our literature search identified no non-randomized controlled trials and four randomized controlled trials of HOP (or COP, its previous name); data from a fifth RCT, submitted for publication, was kindly provided by Lindsay Sheehan, Chicago [46]. All trials had a similar study design (see Table 3 for characteristics of included RCTs). The first trial in Switzerland found short-term benefits on stigma stress, secrecy and disclosure-related distress that were only partly sustained at three-week follow-up [47]. A Californian RCT reported benefits on stigma stress appraisals, self-stigma and, among women, on depression that were mostly sustained at one-month follow-up [48].

**Table 3:** Characteristics of included HOP RCTs

Study	Number of HOP sessions	Comparison condition	Study population (with psychiatric diagnoses, if available)	Setting	% female	Mean age (SD)	Race/ethnicity (if above 5%)	N (HOP, Control)	Assessments after baseline	% Dropout at first post-baseline assessment	Country
Conley et al. 2020 [49]	4 (3 + 1 booster session)	TAU	College students aged 18 or older with self-reported mental illness (diagnoses not specified)	Campuses of three US universities	82	21 (5)	69% White, 18% Asian American, 8% African American	118 (63, 55)	Post; 3-week follow-up (i.e. after the booster session for HOP participants)	8%	USA
Corrigan et al. 2015 [48]	3	TAU	Adults with self-reported mental illness (diagnoses not specified)	Multiple California communities	64	46 (13)	50% White, 27% African American, 21% Hispanic/Latino, 8% Asian, 6% Native American	205 (107, 98; data available from: 51, 75)	Post; 1-month follow-up	39% of randomized participants did not attend groups and/or not complete pre- and post-assessments	USA
Mulfinger et al. 2018 [21]	3	TAU	Adolescents (13–18 years) with mental illness (59% depressive disorder, 17% anxiety disorder)	Inpatient (87%), day clinic (7%) and outpatient (6%) in 3 Dept.s of Child and Adolescent Psychiatry	69	16 (1)	100% White	98 (49, 49)	Post; 3-week follow-up	14%	Germany
Rüsch et al. 2014 [47]	3	TAU	Adults with self-reported mental illness (60% depressive disorder, 20% bipolar dis., 27% schizophrenia)	Outpatient / Community (Zürich)	59	42 (11)	100% White	100 (50, 50)	Post; 3-week follow-up	13%	Switzerland
Sheehan et al. (subm. [46])	3	TAU	Adults with a history of at least 1 lifetime suicide attempt (diagnoses not specified)	Community (Chicago)	39	48 (14)	82% Non-White, 18% White, 11% Hispanic/Latino	38 (19, 19)	Post; 3-week follow-up	8%	USA

*TAU* treatment as usual, *SD* standard deviation, *post* at the end of the HOP program/after the third session, *N* number of randomized participants

An RCT of HOP for adolescents in Southern Germany showed positive effects post intervention and at follow-up on stigma stress, self-stigma, quality of life, depression, recovery and help-seeking attitudes [21]. The effect on quality of life at follow-up was mediated by reduced stigma stress after the intervention. In a preliminary economic analysis, HOP program delivery costs were much lower than average mental health service costs, and HOP was cost-efficient in terms of quality of life gains (appr. €7.000–€20.000 per QALY, depending on whether costs of HOP facilitator training were included [21]). Fourth, an RCT among US college students showed benefits on self-stigma, self-efficacy in terms of disclosing and on resources to cope with stigma; this study was the only one to offer a fourth booster session [49]. The fifth and yet unpublished RCT evaluated the 2Share HOP version among suicide attempt survivors in Chicago and found positive effects on self-stigma and depression after the intervention [46].

We identified one HOP RCT among adults with mental illness, led by Winnie Mak in Hong Kong; it was unpublished and data were not yet available for the meta-analysis (www.2.ccrb.cuhk.edu.hk/registry/public/615/history/2452). Katrina Scior and her colleagues in London, UK, recently completed a pilot study of their HOP version for mental health professionals, but results are yet unpublished (ucl.ac.uk/pals/hop-mhp-project). Jem Bhatt and colleagues in London, UK, found in a feasibility study that their HOP version for people living with dementia appeared helpful based on qualitative participant feedback, but recruitment was challenging [50]. In Lausanne, Switzerland, HOP (“Honnête, Ouvert, Prêt”, in Engl. “Honest, Open, Ready”) was acceptable and feasible for adults with mental illness [51]. One further study from São Paulo, Brazil, among 31 adults with schizophrenia led by Viviane Setti was an uncontrolled pre-post evaluation and therefore not included in our meta-analysis; groups in this study were not peer-led, but run by mental health professionals [52].

### Outcomes for meta-analysis

Two RCTs reported stigma stress (the difference score, see Table 2), but not the underlying subscale scores [21, 47]; two other RCTs only reported the subscale scores, not stigma stress (the difference score [48, 49]). The first RCT had assessed, but not reported, depression after baseline because depressive symptoms were not considered a HOP outcome at that time [47].

To allow a comparison of HOP effects on self-stigma, we chose the only self-stigma measure that was used in all published RCTs, the apply subscale of the Self-Stigma in Mental Illness Scale-Short Form (SSMIS-SF, Table 2); regarding the unpublished 2Share RCT for suicide attempt survivors [46], only the harm subscale of the SSMIS-SF was available which is highly correlated with the apply subscale and has an identical score range [53].

### Meta-analysis of HOP efficacy

**Fig. 1:**
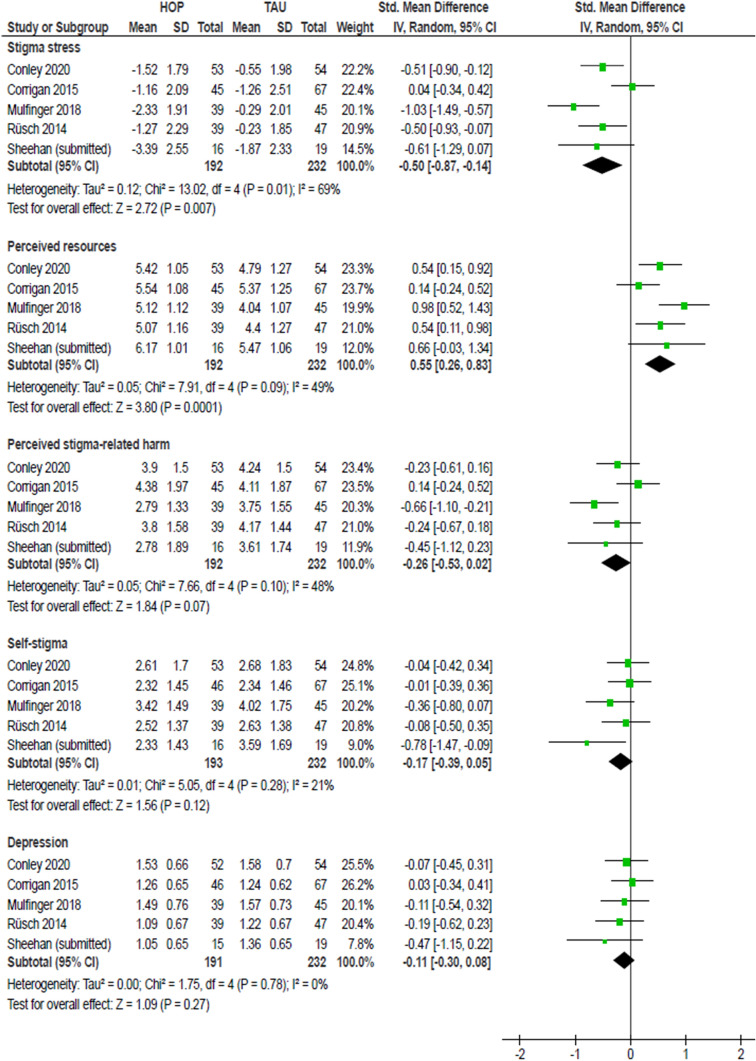
Meta-analysis of HOP effects at the end of the HOP program (post intervention). A negative SMD (to the left) indicates a positive HOP effect, i.e. a reduction of stigma-related variables; except for perceived resources to cope with stigma where an increase (to the right) indicates a positive effect

**Fig. 2:**
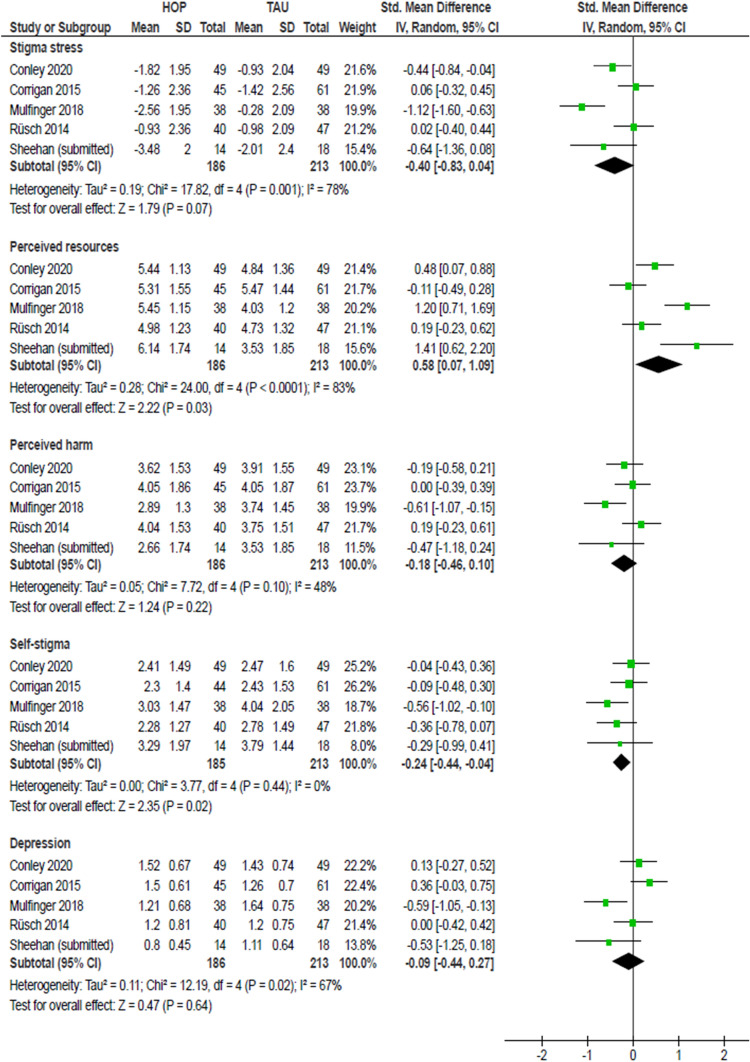
Meta-analysis of HOP effects at 3- or 4-week follow-up. A negative SMD (to the left) indicates a positive HOP effect, i.e. a reduction of stigma-related variables; except for perceived resources to cope with stigma where an increase (to the right) indicates a positive effect

For the meta-analysis of HOP efficacy, we looked at three outcomes: (i) stigma stress, including the subscales of perceived stigma-related harm and of perceived coping resources to clarify whether reduced stigma stress was driven by perceptions of increased coping resources and/or of reduced stigma-related harm; (ii) self-stigma; and (iii) depressive symptoms. For all outcomes, meta-analyses examined the efficacy at the end of the 3-week HOP program (Fig. 1) and, depending on the RCT, at 3- or 4-week follow-up (Fig. 2). For the two subscales of the Stigma Stress Scale, benefit of the HOP program is indicated by *decreased* perceived harm and *increased* coping resources.

Short-term effects at the end of the HOP program showed a positive direction of change, whether or not significant, for all outcomes. Effects were statistically significant for stigma stress with a medium effect size after the intervention (smd = − 0.50, 95%-CI − 0.87 to − 0.14; Fig. 1), but heterogeneity of effects was high (*I*^2^ = 69%). As evident from the effects on the two subscale scores, reduced stigma stress was mainly driven by significantly increased perceived coping resources to deal with stigma (smd = 0.55, 95%-CI 0.26–0.83) and also by non-significantly reduced perceived stigma-related harm (smd = − 0.26, 95% CI − 0.53 to 0.02; Fig. 1). Effects on self-stigma (smd = − 0.17, 95%-CI − 0.39 to 0.05) and depression (smd = − 0.11, 95%-CI − 0.30 to 0.05) at the end of the HOP program were small and statistically not significant, with homogenous effects (Fig. 1).

At follow-up, the meta-analysis revealed a medium effect on stigma stress (smd = − 0.40, 95%-CI − 0.83 to 0.04) which did not reach statistical significance and effects were highly heterogeneous (*I*^2^ = 78%; Fig. 2). Here again, the effect was mainly driven by significantly increased coping resources (smd = 0.58, 95%-CI 0.07–1.09). The small effect of HOP on self-stigma (smd = − 0.24, 95%-CI − 0.44 to − 0.04) was statistically significant at follow-up with no heterogeneity (Fig. 2). There was no significant effect on depressive symptoms (smd = − 0.09, 95%-CI − 0.44 to 0.27; Fig. 2), with high heterogeneity of effects (*I*^2^ = 67%).

## Discussion

This meta-analysis of five HOP RCTs suggests that HOP is a helpful peer-led intervention to support participants with mental illness in their dealing with stigma. HOP reduced the perception of stigma as a stressor. HOP also had some positive effects on self-stigma, while effects on depressive symptoms were less consistent.

One RCT had reported significantly positive effects on stigma stress appraisals [48]. However, in our meta-analysis, we found no significant effect on subscale appraisals or the stigma stress score for that RCT. This is likely due to the fact that HOP participants in that trial reported substantially more perceived stigma-related harm at baseline than the control group (see Fig. 2 in [48]); after baseline, harm levels of HOP participants approached control group levels. In our meta-analysis, only post-baseline scores were taken into account (see “Methods” above), therefore we did not detect a significant intervention effect on stigma stress appraisals for that trial (Figs. 1 and 2).

This meta-analysis has a number of limitations: the number of RCTs was small and there was no (completed) RCT from a low- or middle-income country; only three outcome domains could be compared across all trials; and there was high heterogeneity between studies. Nevertheless, the meta-analysis provides initial evidence for HOP’s efficacy on stigma stress and self-stigma across different target groups and settings.

Future research will need to determine predictors of HOP outcomes. One could speculate, for example, that HOP more effectively reduces depression among women [48] and that HOP may be more effective among young participants whose social networks and disclosure decisions are still more in flux [21]. It should also be examined whether more recent HOP versions that include a fourth booster session [49] have better long-term effects.

### Knowledge gaps

Our findings highlight two major gaps in our knowledge about HOP and its efficacy. First, there are no follow-up data longer than one month after the end of the intervention, and long-term effects are unclear; this information would be important to assess the sustainability of positive effects as well as potential long-term risks for those HOP participants who decide to disclose but may face discrimination later on. Second, the role of actual disclosure decisions is unclear. We do not know whether HOP helps especially those who decide and learn how to disclose strategically; or whether HOP is equally or even more beneficial for participants who choose “empowered non-disclosure”.

### Implications for outcome measurement

It can be expected that HOP has proximal effects on stigma-related variables, such as stigma stress and self-stigma (Table 2). The well-established negative effects of stigma on a person’s well-being explain why HOP may also positively affect more distal outcomes, such as quality of life, recovery and depression (see Fig. 2 in [21]). Therefore future trials should measure proximal (stigma-related) outcomes as well as more distal outcomes. Finally, help-seeking requires some degree of disclosure and HOP may improve help-seeking attitudes [21], possibly mediating social and clinical long-term effects of HOP.

At a more technical level, the assessment and reporting of outcomes in future HOP trials could be improved. First, a joint battery of key outcome measures would allow comparisons across trials (see Table 2 for a start). Second, results of the same scale have been reported inconsistently in different trials. Stigma stress is conceptualized as the difference between perceived harm and perceived coping resources, based on Lazarus’ transactional model of stress and coping [54]. Stigma stress occurs when people feel that stigma-related harm exceeds their coping resources. To assess stigma stress, both appraisals (harm *and* coping resources) need to be taken into account. Rather than both separate appraisals, the key variable to report is the stigma stress difference score, with a range of possible scores from − 6 to + 6 (Table 2). In two trials, this stress difference score was not reported, only the two underlying subscale scores [48, 49]; in one trial, the perceived harm subscale was erroneously referred to as “stigma as a stressor” [49].

### Challenges for HOP evaluation and implementation

There are a number of challenges to high-quality research on HOP and to broadening the evidence base. First, HOP should be implemented and evaluated as a peer-run program. Peers have gone through disclosure decisions themselves and can be role models for participants. Thus, HOP can be considered a structured, manualized, time-limited mutual support group. It is a misunderstanding to call an intervention “HOP” if groups are run by mental health professionals. This happened in the above-mentioned uncontrolled Brazilian study; due to measurement problems, pre–post changes of stigma stress in this study remained unclear [52].

Second, recruitment to HOP studies and to HOP groups can be a challenge. For example, two German trials of HOP versions had to be ended because recruitment was nearly impossible: One for 2Share in a psychiatric inpatient setting (clinicaltrials.gov/ct2/show/NCT03943862), the other as an online version of Starting the Conversation for parents (clinicaltrials.gov/ct2/show/NCT04107714). In our experience, feedback of nearly all HOP participants is very positive, but many who might be troubled by stigma and disclosure decisions do not attend. This may be related to the intrinsic dilemma that by attending a HOP group you disclose your identity, at least to those in the same group. This led Katrina Scior and colleagues in London, UK, to offer HOP groups for mental health professionals in an anonymous online self-help setting (isrctn.com/ISRCTN18418155; Table 1). Another way to address this problem is to turn HOP into an individual intervention in which one peer worker talks to one participant; Melissa Pyle and colleagues in Manchester, UK, are evaluating their 1-to-1 version of HOP, “Let’s Talk”, in an ongoing project (Table 1). Results will help to answer the question whether HOP is feasible and effective outside group settings.

Third, to reduce bias due to selective reporting of positive results, future HOP trials should be pre-registered in publicly accessible trial registries with defined primary endpoints as has become the norm for clinical trials.

### Future developments

In the development of new HOP versions for different target groups and cultural contexts, it will be a challenge to keep the balance between flexibility and adaptation on the one hand, and on the other hand, fidelity to the basis of the joint HOP approach. There is an international HOP steering committee led by Pat Corrigan that tries to address these issues. In terms of evaluation, there is a lack of long-term follow-up assessments and qualitative research about the experience of HOP participants. It is also unclear whether those participants who decide to disclose have better outcomes than those who decide not to disclose.

By supporting strategic and successful disclosure (i.e. telling the right story to the right people at the right time), HOP has the potential to also reduce public stigma. Disclosure that works well for discloser and recipient means positive contact—arguably the most potent strategy to reduce the public stigma of mental illness [55, 56]. However, this is a hypothesis that will not be easily tested empirically.

## Conclusion

Based on the available evidence, HOP as a compact peer-led group program appears to be effective and possibly cost-efficient. It can be offered flexibly for different target groups in different settings. HOP supports participants to handle disclosure decisions and thus reduces stigma’s impact. Research is needed on its long-term effects. To achieve lasting change in a public health sense, HOP should be combined with programs to reduce public stigma for two reasons: First, people with mental illness must not be left alone in dealing with a stigma for which they are not responsible; second, only decreased public stigma will lead to a less prejudiced social environment that facilitates disclosure, positive contact and social inclusion.

## Data Availability

Not applicable as no primary data was collected.
